# Genetic Association and Gene-Gene Interaction Reveal Genetic Variations in ADH1B, GSTM1 and MnSOD Independently Confer Risk to Alcoholic Liver Diseases in India

**DOI:** 10.1371/journal.pone.0149843

**Published:** 2016-03-03

**Authors:** Neelanjana Roy, Debanjali Dasgupta, Indranil Mukhopadhyay, Ankita Chatterjee, Kausik Das, Pradip Bhowmik, Soumyajit Das, Priyadarshi Basu, Amal K. Santra, Simanti Datta, Gopal Krishna Dhali, Abhijit Chowdhury, Soma Banerjee

**Affiliations:** 1 Center for Liver Research, School of Digestive and Liver Diseases, Institute of Post Graduate Medical Education and Research, Kolkata, West Bengal, India; 2 Indian Statistical Institute, Kolkata, India; 3 National Institute of Biomedical Genomics, Kalyani, India; 4 Department of Hepatology, School of Digestive and Liver Diseases, Institute of Post Graduate Medical Education and Research, Kolkata, West Bengal, India; 5 Agartala Government Medical College, Agartala, Tripura, India; 6 Department of Gastroenterology, School of Digestive and Liver Diseases, Institute of Post Graduate Medical Education and Research, Kolkata, India; Saint Louis University, UNITED STATES

## Abstract

Genetic susceptibility is an important modifier of clinical outcome and natural history of progression in Alcoholic liver disease (ALD). While the significance of ethnicity in this evolution is very clear, subtle inter-individual genetic variant(s) might be important and thus we investigated those in an Indian population. Fourteen markers were genotyped within two alcohol metabolism genes [Alcohol dehydrogenase (*ADH*) gene clusters (*ADH1B* and *ADH1C*) and Aldehyde dehydrogenase (*ALDH2*)], one microsomal ethanol oxidizing enzyme cytochrome p450 (*CYP2E1*) and three oxidative stress response (OSR) genes (*MnSOD*, *GSTT1* and *GSTM1*) among 490 Bengali individuals (322 ALD and 168 control) from Eastern and North-Eastern India and validation was performed in a new cohort of 150 Bengali patients including 100 ALD and 50 advanced non-alcoholic steatohepatitis (NASH). Out of 14 genetic variants, carriage of 5 genotypes (rs2066701CC in *ADH1B*, rs1693425TT in *ADH1C*, rs4880TT in *MnSOD* and *GSTT1*/*GSTM1* null, p-value <0.05) were noted significantly higher among ALD patients while inter or intra group gene-gene interaction analysis revealed that addition of risk genotype of any OSR gene enhanced the possibility of ALD synergistically. Multiple logistic regression analysis showed independent association of rs2066701CC, rs4880TT and *GSTM1* null genotype with ALD while lower frequencies of those genotypes in advanced NASH patients further confirmed their causal relation to ALD. Thus these findings suggest that the three variants of *ADH1C*, *MnSOD* and *GSTM1* can be used to identify individuals who are at high risk to develop ALD and may be helpful in proper management of Indian alcoholics.

## Introduction

Alcoholic liver disease (ALD) is one of the fast emerging common causes of chronic liver diseases across the globe. It is the clinical consequences of continuous alcohol over consumption (for e.g., >80gm/day for more than 10 years) [1], which includes reversible fatty liver stage to end-stage cirrhosis through steatohepatitis with or without fibrosis [2]. Although a dose effect linkage has been documented in ALD [3], only 20–40% of chronic alcoholics develop alcoholic hepatitis [4] and 8–20% progresses to cirrhosis [5] implicating the role of certain host genetic factors on the development and progression of ALD. Family, twin and adoption studies have further supported the intermittent relation between genetic determinants and ALD [6–8].

The biological relevance of genetic variations in two major alcohol metabolizing genes, alcohol dehydrogenase (*ADH*) and aldehyde dehydrogenase (*ALDH*) (particularly *ALDH2*) in risk of development of ALD have been extensively investigated [9–10]. Genetic polymorphisms in *ADH* gene, which oxidizes alcohol into less toxic acetaldehyde have exhibited significant association in few of the population based case-control studies while others did not [11]. Similarly, case-control studies with mitochondrial ALDH, which oxidizes acetaldehyde to acetate revealed incongruous results such as ALDH2*2 allele exclusively predispose in Asians [12] and experiences a negative physiological response against alcohol induced tissue damage whereas in another Asian study a significantly increased risk of this genotype was observed among moderate alcohol consumers [13].

Alcohol functions in a myriad of pathways to damage liver in a concerted manner. Genetic variations in *CYP2E1*, which promotes generation of reactive oxygen species (ROS), enhances lipid peroxidation and production of reactive aldehydes with potent pro-inflammatory activities [14] and three enzymes namely Glutathione-S-transferases (*GSTT1 and GSTM1*) and Manganese superoxide dismutase (*MnSOD*) which counteract the actions of reactive oxygen species (ROS) deserve enormous importance in the development of ALD [15]. Deletion polymorphism studies with *GSTM1* and *GSTT1* in ALD also revealed contradictory results [16–17]. Case-control studies with a variation in the codon 16 of the precursor protein of *MnSOD* [alanine (Ala) to valine (Val)] at the –9 amino acid position of the N-terminal signal sequence [18], which alters its correct transport and processing in the mitochondria showed association of Ala/Ala genotype with increased risk of liver cancer, breast cancer and prostate cancer in few population studies [19]. Thus an assessment of the etiological components of alcohol metabolism and oxidative stress pathways and their interaction may offer an alternative in controlling this global problem.

In India, with the socio-economic transition the new population groups vulnerable to alcohol related morbidities is rising rapidly. Although a very few case control association study has been documented in ALD [17,20] from North India, the genetically diverse Indian population needs well designed study to identify the susceptible risk variants as early disease predictor. Here, we have investigated the genetic association of different genes from alcohol metabolism and oxidative stress response pathways with ALD among “Bengalis” from East and North-East region of India. Considering the complexity in ALD, gene-gene interaction was performed to enhance the accuracy for predicting the high-risk individual. Finally, logistic regression analysis was used to identify ALD specific independent risk variants that may facilitate the prediction of ALD risk group.

## Materials and Methods

### Selection of Cases

**Fig 1:**
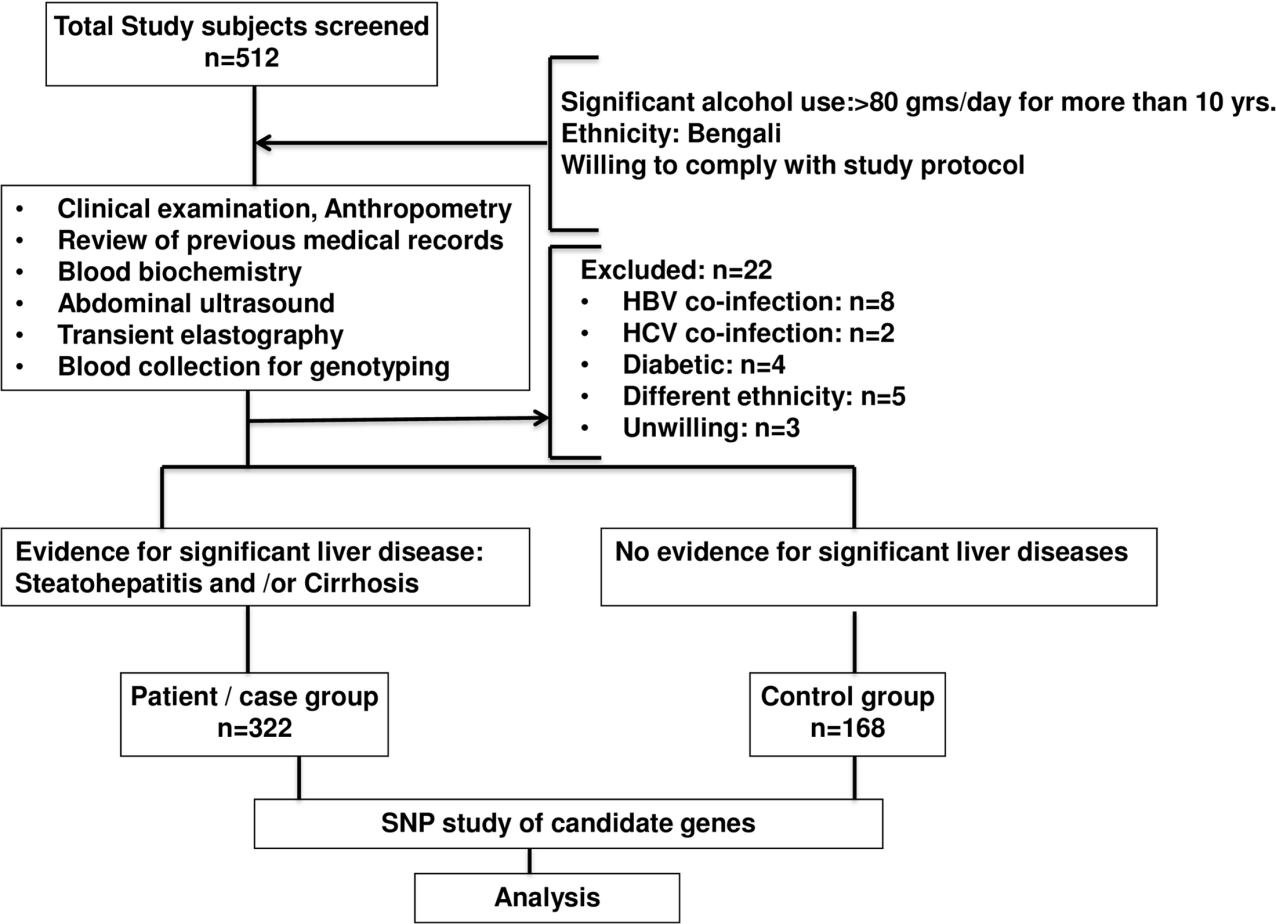
Schematic presentation of study design. An overview on steps followed to identify risk factors for the development of ALD.

A total of 422 consecutive subjects, belonging to a single ethnicity (Bengali), with history of significant alcohol consumption, defined as more than 80 g/day for more than 10 years [21] attending the OPD and indoor of (1) School of Digestive and Liver Diseases, Institute of Post Graduate Medical Education and Research (IPGME&R), Kolkata, India (East) (n_case/KOL_ = 220) and (2) Agartala Medical College, Tripura, India (North-East) (n_case/NE_ = 202) were enrolled for this study. They were assigned to patient group as per predefined inclusion/ exclusion criteria [21] (**Fig 1**). Diagnosis of (a) cirrhosis was based on clinical and radiological parameters (portal hypertension, esophageal/gastric varices and with ascites as decompensated otherwise compensated) and (b) steatohepatitis was based on ultrasonographic evidence of fatty liver and elevated liver enzymes without histological proof of cirrhosis. Hepatocellular carcinoma (HCC) was not detected in any subject included in the study.

### Selection of Controls

A total of 168 Bengali alcoholic control subjects (ALC) from two different regions (n_control/KOL_ = 98 and n_control/NE_ = 70) were enrolled having “significant” alcohol intake but no evidence of liver diseases detected either in biochemical tests (normal liver function tests) or in ultrasonography, endoscopy and/ or in transient elastography [22].

### Exclusion criteria for case and control subjects

Subjects were excluded if they had any of the following criteria (i) HBsAg, anti-HCV or HIV sero-positivity, (ii) presence of diabetes mellitus, (iii) evidence for alternative causes for liver diseases (e.g. Wilson's disease, autoimmune and drug induced liver diseases, etc.), (iv) different ethnicity and (v) unwillingness to comply with the study protocol.

Although “Bengali” patients and controls were included from two different geographical locations, both the groups exhibited similar pattern of allelic distribution. Therefore, two groups were clubbed and articulated as single module.

### Non-alcoholic steatohepatitis patients (NASH)

Histologically verified 50 NASH patients with comparable age, sex and having no other liver diseases were included in the study from Department of Hepatology, IPGME&R, Kolkata.

### Ethical Statement

Institutional ethical review committee of Institute of Post Graduate Medical education and Research has approved the study (No: INST/ISE/1633). Informed written consents were collected from all participants in the study.

### Sample handling and DNA Preparation

The blood specimen was drawn by venipuncture from each participant and stored in two parts–(1) 3ml blood with EDTA for isolation of genomic DNA by salting-out method [23], (2) 2 ml blood subjected to separate serum for biochemical assays and detection of viral markers using commercially available kits from Bayer Diagnostics, India.

### Selection of genes and genetic variants

Four alcohol metabolizing genes *ADH1B*, *ADH1C*, *ALDH2*, *CYP2E1* and three OSR pathway genes *MnSOD*, *GSTM1*, *GSTT1* were selected for this study. A total of 14 SNPs (rs2066701 and rs1229984 of *ADH1B*; rs698, rs1789920, rs1693425 of *ADH1C*; rs441, rs2238151 and rs4648328 of *ALDH2*; rs3813867, rs2031920, rs2031921 of *CYP2E1*; rs4880 of *MnSOD*; null allele of *GSTM1* and *GSTT1)* which were previously reported as ALD associated variants in other population were selected for this study.

### Genotyping

The frequency distribution of allelic variants of the selected SNPs were determined by either restriction fragment length polymorphism (RFLP) (rs2066701/RsaI, rs698/SspI, rs1789920/EcoRI, rs1693425/HaeIII, rs441/HaeIII, rs2238151/HaeIII and rs4648328/RsaI) or sequencing (rs2031920, rs2031921, rs3813867 and rs4880) on ABI Prism 3100 Genetic Analyzer using Big-Dye Terminator v3.1 (Applied Biosystems). Restriction enzymes were purchased from New England Biolabs. The accuracy of the variants detected by RFLP was confirmed for each locus by sequencing of randomly selected 5% of total samples. PCR was performed using locus specific primer **(S1 Table)**. Deletion polymorphism in *GSTT1* and *GSTM1* genes were determined by multiplex PCR.

### Statistical analysis

All the statistical analyses were performed using SPSS (version 10.0) and R software (version 2.0). Associations between alleles/ genotypes and disease occurrence were considered ‘‘strong” and ‘‘statistically significant” where p-value is < 0.05.

#### (a) Allele and Genotype frequency

Initially the quality of each genotype was tested for Hardy-Weinberg equilibrium by using Haploview software. For association analysis, allelic and genotypic frequencies were calculated by direct gene counting method. Considering the major allele in the control population as reference allele 1 and variant allele 2, the associated risk genotypes were evaluated using dominant (11+12 vs. 22), recessive (11 vs. 22) and additive (11 vs. 12 **+**22) genetic model. Benjamini-Hochberg multiple testing correction was employed to control false discovery rate.

#### (b) Gene gene interaction

To determine the influence of more than one variant on disease risk gene-gene interaction study was done by Fishers’ exact test.

#### (c) Multiple Logistic regression analysis

To identify the independent predictor for ALD multiple logistic regression analysis was performed, where all significantly associated variants from univariate analysis and co-variates such as age were considered.

## Results

This study had explored the role of genetic variations in two primary alcohol metabolizing enzymes *ADH* (*ADH1B* and *ADH1C*) and *ALDH* (*ALDH2*); CYP2E1 of microsomal ethanol oxidizing system (MEOS) and three oxidative stress response pathway genes (*MnSOD*, *GSTT1* and *GSTM1*).

### Demographic, clinical and biochemical characteristics of study subjects

A total of 512 subjects were included in the discovery cohort of the study, out of which 490 subjects (control 168, cases 322) had achieved all inclusion criteria and included for study to sustain a homogenous clinical feature which provided the power of the study as 90%. Case and control groups were clustered differentially according to their baseline characteristics **(Table 1)**.

**Table 1:** Demographic, biochemical and clinical features of the study population.

Variables	Total ALD Patients^1^ (n = 322)	Steatohepatitis with fibrosis /Compensated Cirrhosis^2^ (n = 127)	Decompensated Cirrhosis^3^ (n = 195)	ALC^4^ (n = 168)	*p-value (1vs4)	*p-value (2vs3)	*p-value (2vs4)	*p-value (3vs4)
**Epidemiology**								
Gender (M:F)	322:0	127:0	195:0	168:0	-	-	-	-
Age, Years	44.98	45.04	44.02	41.46	**NS**	**NS**	**NS**	**NS**
(Mean±SD)	± 10.24	± 10.55	±9.05	±10.07				
Child-Pugh	7.85	6.50	8.10	-	**-**	**<0.01**	**-**	**-**
Score (Mean±SD)	±0.885	± 0.502	± 0.611					
BMI (kg/m^2^),	20.51	20.11	19.76	23.51	**<0.001**	**NS**	**<0.00**	**<0.00**
(Mean±SD)	±3.03	±3.01	±3.67	±3.73			**1**	**1**
Ascitis	136/322	0/127	136/195	0/169	**-**	**-**	**-**	**-**
Total alcohol	13808.89	12068.89	12808.89	12029.	**NS**	**NS**	**NS**	**NS**
consumption(gms) (Mean±SD)	±6749.57	±5849.29	±6746.57	37 ± 3859.26				
Liver Stiffness Measurement (kPa); (Mean±SD)	ND	ND	ND	7.46±3.45	**-**	**-**	**-**	**-**
**Laboratory results**								
Total Bilirubin(mg/dl) (Median, Range)	4.1 (0.9–26.6)	3.31 (0.9–12.4)	4.1 (0.9–26.6)	0.9 (0.9–1.8)	**<0.0001**	**<0.005**	**<0.001**	**<0.001**
Albumin(g/dl) (Mean±SD)	3.35 ± 0.826)	3.384 ± 0.8275	3.295 ± 0.7631	4.09 ± 0.556	**<0.005**	**NS**	**<0.05**	**<0.05**
ALT(u/l)(Median, Range)	63 (11–756)	69.66 (44–756)	63 (11–357)	29 (18–46)	**<0.0001**	**NS**	**<0.001**	**<0.001**
AST(u/l) (Median, Range)	61 (19–1125)	63.00 (19–1125)	64.50 (19–514)	33 (13–44)	**<0.0001**	**NS**	**<0.001**	**<0.001**
Alkaline Phosphatase (IU/L) (Median, Range)	172 (30–397)	168 (119–346)	178 (130–397)	119 (92–148)	**<0.0001**	**NS**	**<0.001**	**<0.001**

* *p-value* of Student’s t test; SD = standard deviation; NS = not significant; “-” indicates not applicable.

### Non-coding variant rs2066701 in *ADH* gene is strongly associated with ALD

**Fig 2:**
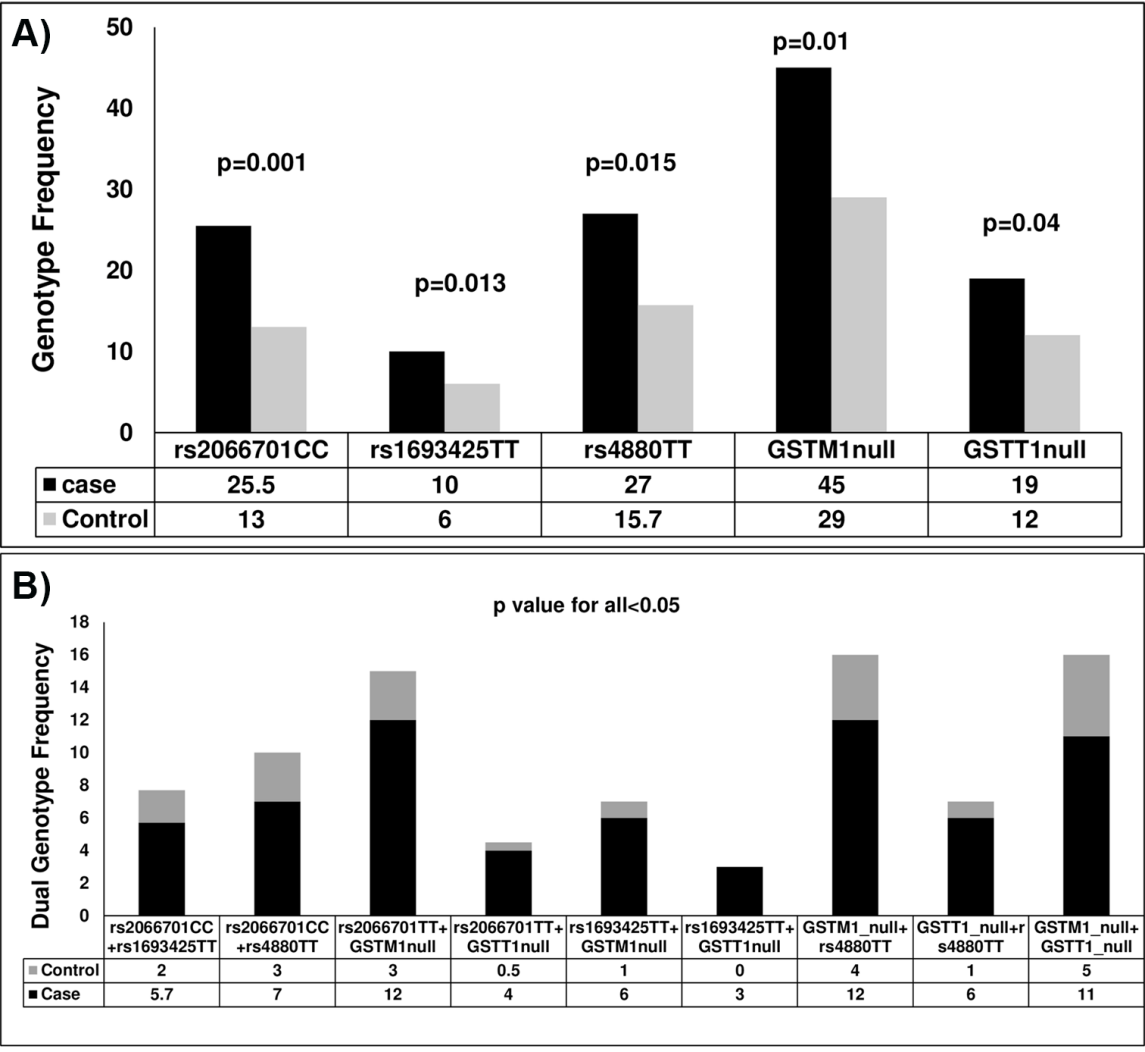
Distribution pattern of prevalent genotypes in ALC and ALD. (A) Risk genotypes at polymorphic loci of *ADH1B*, *ADH1C*, *MnSOD*, *GSTT1* and *GSTM1* assocaited with ALD and (B) Combination of risk genotypes exhibit significant gene-gene interaction in ALD. p<0.05 was considered as significant.

Except rs1789920 in *ADH1C*, all tested variants corresponded to the Hardy-Weinberg equilibrium. Frequency of minor allele “C” (rs2066701) in ADH1B and “T” (rs1693425) in ADH1C gene was significantly low in control subjects than case (0.36 vs. 0.49 and 0.19 vs. 0.23, corrected p-values 0.04 and 0.06 respectively) (**S2 Table**). Hence, distribution of only *ADH1B* genotypes (TT/CT/CC) at rs2066701 loci was found significantly different in ALD from ALC (27/48/26 vs. 40/47/13, p-value after B-H correction = 0.004) (**S3A Table**). In univariate analysis higher frequencies of CC genotype at rs2066701 loci of *ADH1B* (p = 0.0001) and TT at rs1693425 loci of ADH1C (p = 0.013) in ALD patients had confirmed their association with increased risk of ALD with respect to TT and CC genotypes in ALC respectively (**Fig 2A and S3A Table**). No significant alteration in the distribution of genotypes of missense variants at rs698 loci of *ADH1C;* rs2238151, rs4648328 and rs441 in *ALDH2* were observed while rs1229984 loci of *ADH1B* was monomorphic (**S3B Table**).

### Three genotypes of anti-oxidative enzymes *GSTT1/GSTM1* and *MnSOD* are overrepresented among ALD patients

As ethanol promotes oxidative stress by inducing ROS generation and decreasing cellular defence mechanisms, genetic variations in *CYP2E1*, *GSTT1*, *GSTM1* and *MnSOD* were studied. Three loci of *CYP2E1* were monomorphic in the studied population.

Low null allele frequency of *GSTM1* and *GSTT1* in ALC (**S2 Table**) and significantly high null genotype frequency of *GSTM1* in ALD (45% and 19% vs. 29% and 12%; p = 0.004, p = 0.07 respectively) implicating strong association of GSTM1 null variant with disease (**Fig 2A and S3A Table**).

Again the genotype frequencies of Ala/Ala (CC), Ala/Val (CT) and Val/Val (TT) at rs4880 loci of *MnSOD* gene were 33.3%, 51%,16% in ALC and 27%, 46%, 27% in ALD (p = 0.015). Hence total frequency of valine allele (T) was higher among ALD (50%) than ALC (41%) indicating individuals with TT genotype are more prone to development of disease **(Fig 2A and S3A Table)**.

### Pairwise interactions between genetic variants and risk of ALD

Epistasis or gene-gene interaction is an important component of a complex multi-factorial disease. Out of 14 selected variants in *ADH*, *ALDH*, *CYP2E1*, *MnSOD*, *GSTT1* and *GSTM1;* 5 variants were found to be significantly over represented among ALD patients. We explored all possible interactions between genotypes using a regression approach considering double non-risk genotype (11 and 12) at both the loci as reference and compared with either single risk genotype (22) at one locus or double risk genotype (22 and 22) at two loci among ALD and control subjects. Fifteen combinations with double risk genotype at 5 loci showed significant association with ALD. In ALD patients, genotype CC of rs2066701 in *ADH1B* and TT of rs1693425 in ADH1C significantly co-occur more with null genotype of both *GSTT1* and *GSTM1* (4%, 3% and 12%, 6%) compared to control (0.5%, 0% and 3%, 1%) (p<0.05) whereas only rs2066701CC co-exist with rs4880TT variant of *MnSOD* in ALD (7% vs 3%, p = 0.043) (**Fig 2B, S4C Table**) implicating that those loci might interact to increase the risk of development of ALD. In addition, co-occurrence of CC of rs2066701 in *ADH1B* and TT of rs1693425 of *ADH1C* enhanced the risk of the disease (6% in ALD vs. 2% in ALC, p = 0.032) (**Fig 2B, S4A Table**) although the frequency of co-exixtence was quite low. Interestingly, the co-existence of either defective variants of *GSTT1* or *GSTM1* or combination of each with rs4880TT of *MnSOD* increased the risk of ALD synergistically (p<0.05 for all) **(Fig 2B, S4B Table**). It is noteworthy to be mentioned that very low frequency in carriage of triple risk genotype (*GSTT1*, *GSTM1* and *MnSOD*) among ALD patients (11/275, 4% only) was observed and as expected it was completely absent among control subjects (0/153) (data not shown). Taken together, the results of these analyses revealed that the null genotype of oxidative stress response gene *GSTM1* and CC genotype at rs2066701 of *ADH1B* in combination with any other risk variant showed strong association with the increased susceptibility to ALD.

### Multiple logistic regression analysis to identify the independent risk loci

No strong linkage disequilibrium (LD) was observed among 5 genetic variants identified as associated risk factor in this study (data not shown) indicating independent involvement of multiple loci with the development of ALD. To identify the independent risk factor multiple logistic regression analysis (MLRA) with all variables having p-value <0.05 in previous analysis were performed. MLRA revealed that only three variants, null genotype of *GSTM1* (p = 0.005), rs4880 in *MnSOD* (p = 0.008) and rs2066701 in *ADH1B* (p = 0.01) might function as independent risk factor for the development of ALD **(Table 2)**. Stratification of these data in a new cohort would provide more tangible clues to disease management.

**Table 2:** Multiple logistic regression analysis to determine the possible risk factors for development of Alcoholic liver diseases.

Loci ID	OR, 95% CI	p value
rs2066701CC	1.55(1.11–2.16)	**0.01**
rs1693425TT	1.13(0.77–1.66)	0.52
rs4880TT	1.5(1.11–2.03)	**0.008**
GSTM1_null	1.88(1.21–2.93)	**0.005**
*GSTT1_null*	1.86(0.98–3.55)	*0*.*06*

p<0.05 was considered as significant and italics represented the marginal significant value.

### Relation of rs2066701CC in *ADH1B*, rs4880TT of *MnSOD* and *GSTM1* null genotype with disease severity

**Fig 3:**
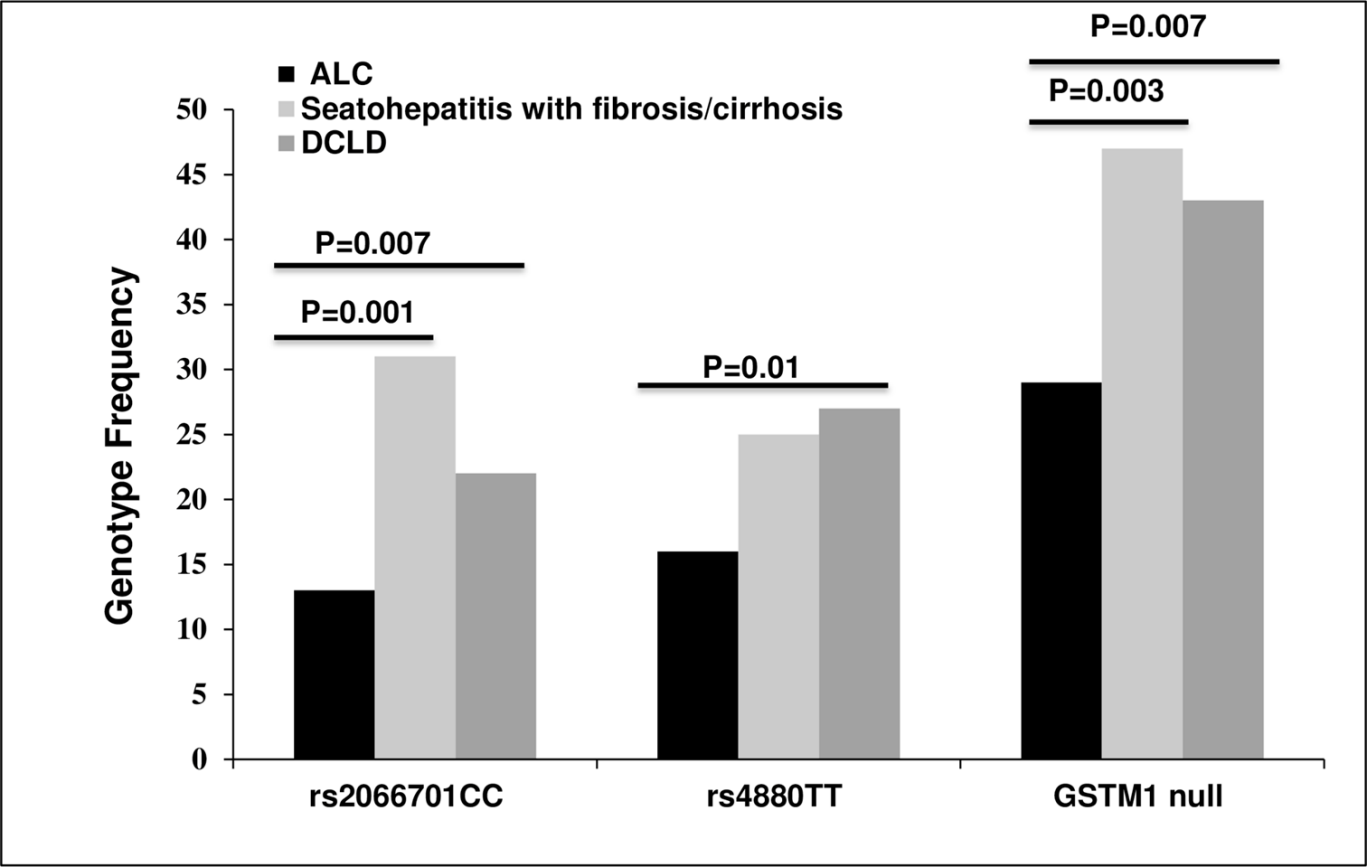
Determination of risk factors associated with disease stages. Risk genotypes were validated in steatohepatitis (with fibrosis/ compensated cirrhosis) and decompensated chronic liver disease (DCLD) stages.

To determine the association of three genetic risk factors with the progression of liver diseases, ALD patients were subgrouped as steatohepatitis with liver fibrosis/ compensated cirrhosis and decompensated chronic liver disease (DCLD) or cirrhosis depending on clinical parameters as described in methods and compared with ALC. It revealed that frequencies of subjects with rs2066701CC and *GSTM1* null genotype were significantly higher in both the disease groups compared to control (p<0.05) whereas rs4880 TT was significantly more only in DCLD (p = 0.01) **(Fig 3, S5 Table).**

### High frequencies of rs2066701CC in *ADH1B*, rs4880TT of *MnSOD* and *GSTM1* null genotypes were only observed in ALD not in NASH patients

**Fig 4:**
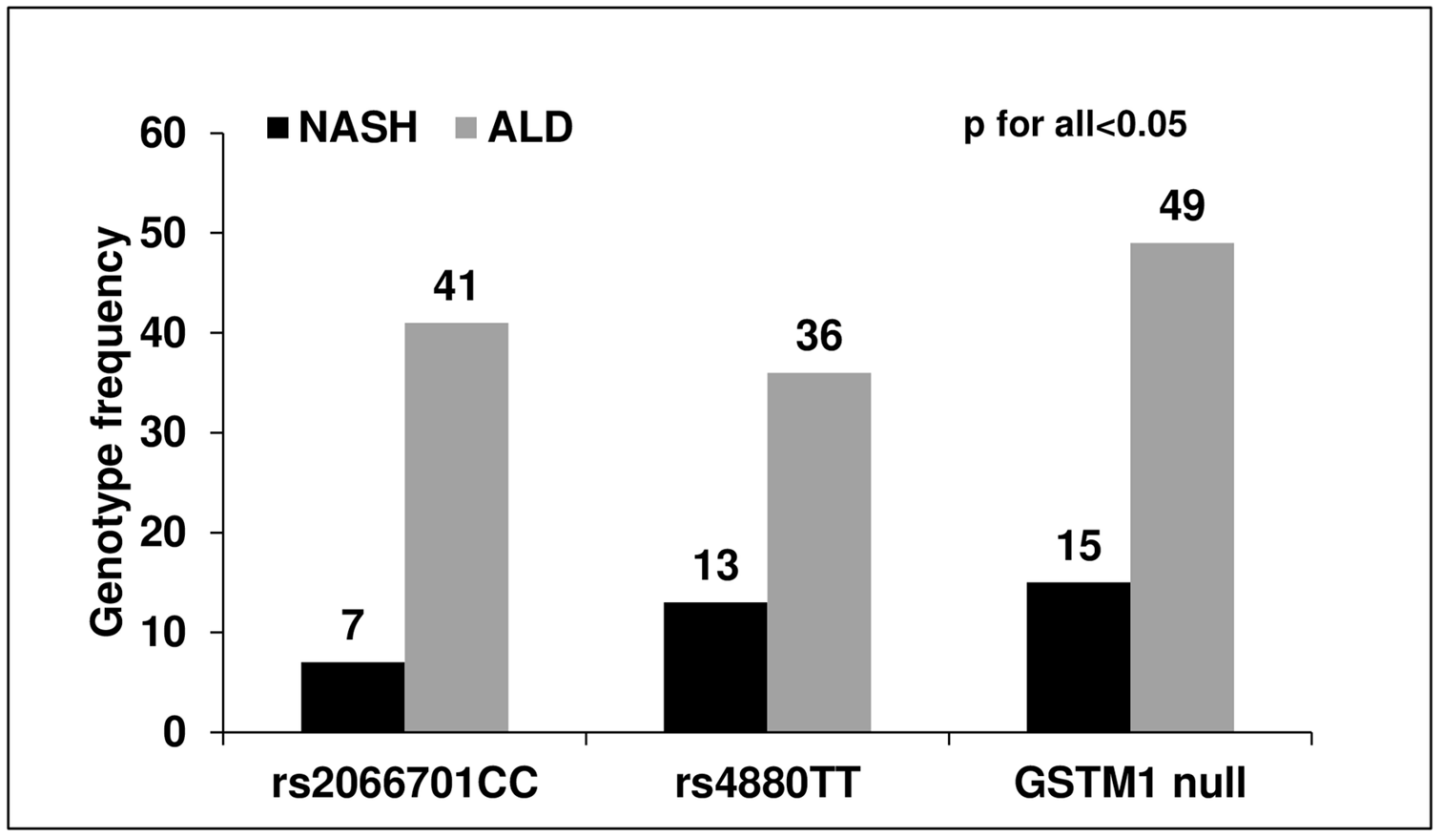
Validation of risk factors in a new cohort of patients. **Distribution of** independent risk factors (rs2066701CC, rs4880TT and GSTM1 null genotype) in a new cohort of ALD and NASH patients were determined.

Although studying the mechanism of genetic variants in the development of ALD were beyond the scope of the current study, but to further elucidate their importance, genotype frequencies at those loci were determined in a cohort of NASH patients, which was included as an independent aetiology in the development of liver diseases and compared with new cohort of ALD patients. The baseline demographic and biochemical characteristics of ALD and NAFLD cohort were shown in **S6 Table**. After adjustment of age, frequencies of CC genotype of rs2066701 (41% vs. 7%), TT of rs4880 (36% vs. 13%) and null *GSTM1* (49% vs. 15%) were found significantly (p<0.001) higher in patients with ALD than NASH suggesting their contribution in the development of ALD **(Fig 4)**.

## Discussion

Enormous socio-cultural, geographical, linguistic and biological diversities among different Indian populations provided an excellent platform for genetic study of human diseases. In this study, among 14 ALD associated genetic variants, 6 variants such as rs2066701 in *ADH1B*; rs1789920 and rs1693425 in *ADH1C*; rs4880 in *MnSOD* and null genotypes of *GSTM1*, *GSTT1* were overrepresented among “Bengali” ALD patients. To our surprise, distribution patterns of genetic variants were almost similar among “Bengalis” enrolled from two geographically distinct regions implicating the least environmental influence on genetic constitution of Bengalis in NE region who were migrated from either Eastern India or Bangladesh. The regression analysis revealed that specifically *GSTM1* played a significant role in the development of the disease. Carriage of *GSTM1* null and rs2066701CC genotype of *ADH1B* were found to be associated with increased risk of progression of ALC to decompensated cirrhosis. Although few case control studies from North India have identified ALD associated markers [17,20] but to the best of our knowledge this is the first systemic study from India considering the gene-gene interaction and multiple logistic regression analysis to identify independent predictors for ALD. rs2066701CC of *ADH1B*, rs4880TT of *MnSOD* and *GSTM1* null genotype were identified as independent risk factor for the development of ALD and failure to display any significant correlation in NASH has further suggested their association with ALD. Hence, those three genetic variants might be considered in combination of the traditional clinical diagnostic factors to determine the disease earlier.

Among the 7 human *ADH* gene loci, two class I *ADH* genes are quite polymorphic with three functional alleles existing for *ADH1B* [ADH1B*1 (Arg^48^Arg^370^), ADH1B*2 (his^48^Arg^370^) and ADH1B*3 (Arg^48^Cys^370^)], two for *ADH1C* genes [ADH1C*1 (Arg^272^Ile^350^) and ADH1C*2 (Gln^272^Val^350^)]. ADH1B*2 allele codes for a higher activity of the enzyme in ethanol oxidation with increased formation of acetaldehyde and ROS and is highly prevalent among neighbouring East-Asians such as Chinese, Japanese and Korean whereas it is least common among Indians [24–26]. A recent meta-analysis study showed insignificant correlation between ALD and *ADH1B* polymorphism [2]. Again ADH1C*1(rs698), which encodes for alcohol metabolism enzyme with high activity, has consistently found protective in alcoholic individuals in Chinese and Korean population [27–28], but found to be associated with increased risk of ALD among North Indian alcoholics [20], but failed to show any association in Eastern Indian alcoholics in this study implicating a strong genetic diversity exist among different Indian populations and hence, the data is inconclusive. Interestingly, one non-coding variant of *ADH1B* (rs2066701) and two of *ADH1C*, rs1693425 (synonymous variants) and rs1789920 identified with significant association to the increased risk of ALD than ALC. Intronic variants may show association for being in linkage disequilibrium with a causal variant and also if situated at the beginning of an intron may result in alteration of the protein by affecting the splicing phenomenon of the nascent mRNA. rs1789920 which is present at the beginning of intron 2 of *ADH1C* may have an important role during the splicing phenomenon.

Out of nineteen putative functional genes that encodes for *ALDH* enzymes, *ALDH2* (mitochondrial isozyme) is highly expressed in the liver and stomach and it has a very high affinity for alcohol detoxification by acetaldehyde oxidation [20,27,28]. Defective *ALDH2* exhibits accelerated risk of progression of gastrointestinal cancers such as gastric cancer, esophageal cancer and colon cancer but its role in the liver, the major organ of alcohol metabolism, is still controversial [29–30]. In this study, we have genotyped three intronic polymorphic sites of *ALDH2* gene (rs2238151, rs4648328 and rs441) but none of them showed sigificant association with ALD among “Bengalis” which is similar to the previous observation by Bhaskar *et al* with six ethnic populations from four linguistic groups in India [31].

Chronic alcohol intake induces the level and activity of *CYP2E1* and generates acetaldehyde and free radicals to induce progression of liver diseases to cirrhosis. The 5’-flanking region of *CYP2E1* gene is highly polymorphic at two non-coding loci, rs3813867 and rs2031920 [3–4]. These two loci are in complete LD (D’ = 1.0) (data not shown) and the related haplotype CYP2E1*5B is associated with higher transcription of *CYP2E1*. Significant association of this variant with ALD and other liver diseases has been documented in Oriental [32] and North Indian population [33] but not in Caucasians [34]. These loci were monomorphic in Bengali population from east and NE India.

The oxidative stress generated in alcoholics is neutralized by two important enzymes, glutathione-s-transfereases (*GSTT1* and *GSTM1*) and superoxide dismutases. Consistent with the North Indian study [17,20], null genotype of *GSTM1* gene and coding variant of *MnSOD* (rs4880Ala) with lower enzyme activity were observed with significantly higher frequencies among ALD in this study. Association of deletion variant of *GSTM1* with ALD has been observed in several studies [35]. However, negative association of this null mutant has been also detected in few studies [36]. *GSTM1* plays critical role in liver diseases as supported by our gene-gene interaction data where cumulative effect of this null variant has been observed with *GSTT1* null genotype, rs4880 of *MnSOD* and rs2066701 of *ADH1B*. Again, Alanine to Valine change in the 9^th^ position of mitochondrial target sequence of the single mitochondrial superoxide scavanger *MnSOD* which alters its amphiphilic helical structure crucial for transport and processing of mitochondrial protein varies among ethnic groups. Although a controversial data is available in the literature, a very few studies documented Valine as risk factor against a large data available for Alanine as effective variant which spontaneously dismutase superoxide to peroxide and oxygen [18–19]. Increased peroxide negatively sensitizes cell to *TNF-α* mediated apoptosis whereas Valine variant inhibits entrance of *MnSOD* to inner membrane and enhances programmed cell death. Furthermore, our logistic regression analysis revealed significant association of null genotype of *GSTM1* and rs4880TT of *MnSOD* with severity of liver diseases.

So far, we have discussed that these polymorphic changes in different associated genes either single or in combination may increase susceptibility to development of ALD. Although determination of the multitude of variants associated with a complex genetic disease is difficult but a multiple logistic regression analyis with all other significantly altered parameters emphasized that null genotype of *GSTM1*, rs4880TT of *MnSOD* and rs2066701CC of *ADH1B* might be independently predisposed to the development of ALD. Moreover, lower frequencies of those risk genotypes in NASH patients further provided strong evidence for their association with ALD.

In conclusion, the current study confirms that the genetic variants in *ADH* and two oxidative stress response genes (*GSTM1* and *MnSOD)* are independently associated with the development of ALD among Bengali alcoholics. A genetic interaction amongst them also deserves importance in disease development. As two genotypes in oxidative stress response genes were also found in significantly higher frequencies among North Indian alcoholics, these genetic variants may serve as powerful predictor of ALD in advance and hence, these markers can be useful in the management of Indian alcoholics. Further stratification in a new cohort of patients and powerful complementary mechanistic studies to determine the physiological and pathophysiological role of those variants in liver disease development has been initiated which may help to improve diagnostic and therapeutic strategies for ALD patients.

## Supporting Information

S1 Table Primer sequences used in PCR product analysis. (DOC)

S2 Table Allelic association of Alcohol metabolism and oxidative stress related genes with Indian ALD patients. (DOC)

S3 Table (a)Genotypic association of the Alcohol metabolism and oxidative stress related genes in Indian ALD patients. (b): Distribution of genotypes at different loci, which were either monomorphic or not significant in Bengali population of India.
(DOC)

S4 Table (a)Interaction between rs2066701 of ADH1B and rs1693425 of ADH1C gene. (b): Interactions between anti-oxidative genes GSTT1, GSTM1 and rs4880 of MnSOD. (c): Inter gene interaction between ALD metabolism and oxidative stress related genes.
(DOC)

S5 Table Univariate analysis of genotypes with severity of liver diseases. (DOC)

S6 Table Demographic, biochemical and clinical features of the validation cohort (ALD and NASH patients). (DOC)

## Abbreviations

ADH: Alcohol dehydrogenase
Ala: Alanine
ALC: Alcoholic control
ALD: Alcoholic Liver Diseases
ALDH: Aldehyde dehydrogenase
ALKP: Alkaline phosphatise
ALT: Alanine aminotransferase
AST: Aspartate transaminase
DCLD: Decompensated chronic liver disease
GSTT1/ GSTM1: Glutathione S- Transferases
HBsAg: Hepatitis B virus surface Antigen
HCV: Hepatitis C virus
HIV: Human immunodeficiency virus
LD: Linkage disequilibrium
MLRA: Multiple logistic regression analysis
MnSOD: Manganese superoxide dismutase
NASH: Non-alcoholic steatohepatitis
Val: Valine
