# Supplementary material for: Genetic Association and Gene-Gene Interaction Reveal Genetic Variations in ADH1B, GSTM1 and MnSOD Independently Confer Risk to Alcoholic Liver Diseases in India
Source: PLoS One. 2016 Mar 3;11(3):e0149843. doi: 10.1371/journal.pone.0149843 (PMC4777485; doi:10.1371/journal.pone.0149843)
Supplement: S1 Table — (DOC) [file pone.0149843.s001.doc]

**Table S1:** Primer sequences used in PCR product analysis.

| Candidate Genes | Genetic variants ID | Primer Sequences (5’ to 3’) |
| --- | --- | --- |
| ADH1B | rs1229984 | ATTCTAAATTGTTTAATTCAAGAAG ACTAACACAGAATTACTGGAC |
|  | rs2066701 | ATATTTATTTTACCCTAAACTTATG  GAGCTAAAACATACTTTGGATAG |
| ADH1C | rs698 | TTGTTTATCTGTGATTTTTTTTG T  CGTTACTGTAGAATAC AAAGC |
|  | rs1789920 | TTGCACCTCCTAAGGCTC  TCTAATGCAAATTGATTGTGA AC |
|  | rs1693425 | TGAGTTTGCACATTAGTTATGG  TGCTCTCAGTTCTTTCT GGG |
| ALDH2 | rs441 | AAATATTGCTCTAGGCCAGG C  TGGGAATTCTAAATGGGACGG |
|  | rs2238151 | GTGAAGGTTGGGCAGGGCAGAAT  ATCAAGGCGAGGGGCAGTCAT C |
|  | rs4648328 | TGCCTCAGCCTCCCCACTA  ACCATGTCCAAATCCACCA G |
| CYP2E1 | rs3813867 | CCAGTCGAGTCTACATTGTCA TTCATTCTGTCTTCTAACTGG |
|  | rs2031920 | CCAGTCGAGTCTACATTGTCA  TTCATTCTGTCTTCTAAC TGG |
|  | rs2031921 | CCAGTCGAGTCTACATTGTCA TTCATTCTGTCTTCTAACTGG |
| MnSOD | rs4880 | CAGCCCAGCCTGCGTAGACGG  GCGTTGATGTGAGG TTCCAG |
| GSTM1 | Null | GAACTCCCTGAAAAGCTAAAGC  GTTGGGCTCAAATATACGGTGG |
| GSTT1 | Null | TCACCGGATCATGGCCAGCA  TTCCTTACTGGTCCTCACATCTC |
