# Supplementary material for: Genetic Association and Gene-Gene Interaction Reveal Genetic Variations in ADH1B, GSTM1 and MnSOD Independently Confer Risk to Alcoholic Liver Diseases in India
Source: PLoS One. 2016 Mar 3;11(3):e0149843. doi: 10.1371/journal.pone.0149843 (PMC4777485; doi:10.1371/journal.pone.0149843)
Supplement: S2 Table — (DOC) [file pone.0149843.s002.doc]

**Table S2:** Allelic association of Alcohol metabolism and oxidative stress related genes with Indian ALD patients

| **Gene Name** | **Locus ID** | **Functional consequence** | **Global MAF** | **MAF** | | **p-value (After B-H correction)** |
| --- | --- | --- | --- | --- | --- | --- |
|  |  |  |  | **Case** | **Control** |  |
| ADH1B | rs1229984  rs2066701 | missense (His48Arg)  Intron variant | C>A(0.16)  C>T (0.45) | 0 0  0.49 (C) 0.36(C) | | -  0.04 |
| ADH1C | rs698  rs1789920  rs1693425 | missense (Ile349Val)  Intron variant  Synonymous | A>G (0.24)  A>C (0.18)  C>T (0.261) | 0.25 0.23  0.20 0.13  0.23 0.19 | | -  -  - |
| ALDH2 | rs441  rs2238151  rs4648328 | Intron variant  Intron variant  Intron variant | T>C (0.319)  T>C (0.223)  C>T (0.20) | 0.23 0.20  0.32 0.30  0.04 0.04 | | -  -  - |
| CYP2E1 | | rs3813867 | | --- | | rs2031920  rs2031921 | | Upstream variant  Upstream variant  Upstream variant | G>C (0.08)  C>T (0.06)  T>C (0.08) | 0 0  0 0  0 0 | | -  -  - |
| MnSOD | rs4880 | missense (Val16Ala) | T>C (0.469) | 0.5(T) 0.41((T) | | - |
| GSTM1 | GSTM1 |  | Null allele | 0.45 0.29 | | 0.003 |
| GSTT1 | GSTT1 |  | Null allele | 0.19 0.12 | | - |

p<0.05 was taken as significant. “-“ indicates either insignificant or not available.
