# Supplementary material for: Genetic Association and Gene-Gene Interaction Reveal Genetic Variations in ADH1B, GSTM1 and MnSOD Independently Confer Risk to Alcoholic Liver Diseases in India
Source: PLoS One. 2016 Mar 3;11(3):e0149843. doi: 10.1371/journal.pone.0149843 (PMC4777485; doi:10.1371/journal.pone.0149843)
Supplement: S3 Table — (a)Genotypic association of the Alcohol metabolism and oxidative stress related genes in Indian ALD patients. (b): Distribution of genotypes at different loci, which were either monomorphic or not significant in Bengali population of India. (DOC) [file pone.0149843.s003.doc]

**Table S3a.** Genotypic association of the Alcohol metabolism and oxidative stress related genes in Indian ALD patients

| **Gene name** | **Locus ID** | **Genotype** | **ALD** | **ALC** | **p-value (After B-H correction)** | ***Adjusted OR,**  **95% CI** | **p-value** |
| --- | --- | --- | --- | --- | --- | --- | --- |
|  |  |  |  |  |  |  |  |
| ADH1B | rs2066701 | T/T  C/T  C/C | n=305(%) | n=163(%) | **0.004** | Ref  1.512 (0.99-2.32)  2.81(1.58-4.99) | 0.063  **0.0001** |
|  |  |  | 82 (27)  145 (48)  78 (26) | 65 (40)  76 (47)  22 (13) |  |  |  |
| ADH1C | *rs1789920*  rs1693425 | A/A  C/A  C/C | n=312(%) | n= 166(%) | 0.041 | Ref  0.935(0.6-1.46)  17.901(2.41-132.8) | 0.820  **0.0001** |
|  |  |  | 213 (68)  68 (22)  31(10) | 123(74.1)  42(25)  1(0.6) |  |  |  |
|  |  | C/C  C/T  T/T | n=277(%) | n=158(%) | 0.149 | Ref  1.383 (0.91-2.11)  3.081 (1.23-7.73) | 0.138  **0.013** |
|  |  |  | 149(55)  101(35)  27(10) | 102(71)  50(23)  6(6) |  |  |  |
| MnSOD | rs4880 | C/C  C/T  T/T | n=279(%) | n=153(%) | 0.07 | Ref  1.16(0.74-1.82 )  2.12 (1.19-3.80) | 0.562  **0.015** |
|  |  |  | 74 (27)  131 (46)  74 (27%) | 51 (33.3)  78 (51)  24 (16) |  |  |  |
| GSTM1 | Null allele | Wild Type  Null | n=314(%) | n=168(%) | **0.004** | Ref  1.949(1.305-3.08 ) | **0.001** |
|  |  |  | 172 (55)  138 (45) | 119 (71)  49 (29) |  |  |  |
| GSTT1 | Null allele | Wild Type  Null | n=314(%) | n=168(%) | 0.07 | Ref  1.78(1.03-3.07) | **0.04** |
|  |  |  | 253(81)  61(19) | 148 (88)  20(12) |  |  |  |

p<0.05 was taken as significant.

**Table S3b:** Distribution of genotypes at different loci, which were either monomorphic or not significant in Bengali population of India.

| **Gene name** | **Locus ID** | **Genotype** | **Case** | **Control** |
| --- | --- | --- | --- | --- |
|  |  |  |  |  |
| ADH1C  (Single variant) | rs698 | A/A  A/G  G/G | n=290 | n=157 |
|  |  |  | 164(68%)  107(37%)  19(6.5%) | 91(60%)  59(37%)  7(5%) |
| ALDH2  (Single variant) | rs441  rs2238151  rs4648328 | T/T  T/C  C/C | n=310 | n=167 |
|  |  |  | 185 (60%)  107 (34%)  18 (6%) | 104 (62%)  56 (34%)  7 (4%) |
|  |  | C/C  C/T  T/T | n= 308 | n= 161 |
|  |  |  | 147 (48%)  121 (39%)  40(13%) | 82 (51%)  60 (37%)  19(12%) |
|  |  | C/C  C/T  T/T | n=174 | n=108 |
|  |  |  | 109(63%)  58(33%)  7(4%) | 71(66%)  32(30%)  5(4%) |
| ADH1B  (Monomorphic) | rs122998 | A/A  C/T  T/T | **n=50** | **n= 50** |
|  |  |  | 50 (100%)  0 (0%)  0 (0%) | 50 (100%)  0 (0%)  0 (0%) |
| CYP2E1  (Monomorphic) | rs3813867 and rs2031920  rs2031921 | c1/c1  c1/c2  c2/c2 | n=177 | n=107 |
|  |  |  | 168(94%)  9 (5%)  0 (0%) | 106 (99%)  1(1%)  0 (0%) |
|  |  | T/T  C/T  C/C | n=177 | n=107 |
|  |  |  | 168(94%)  9 (5%)  0 (0%) | 106 (99%)  1(1%)  0 (0%) |
