# Supplementary material for: Genetic Association and Gene-Gene Interaction Reveal Genetic Variations in ADH1B, GSTM1 and MnSOD Independently Confer Risk to Alcoholic Liver Diseases in India
Source: PLoS One. 2016 Mar 3;11(3):e0149843. doi: 10.1371/journal.pone.0149843 (PMC4777485; doi:10.1371/journal.pone.0149843)
Supplement: S4 Table — (a)Interaction between rs2066701 of ADH1B and rs1693425 of ADH1C gene. (b): Interactions between anti-oxidative genes GSTT1, GSTM1 and rs4880 of MnSOD. (c): Inter gene interaction between ALD metabolism and oxidative stress related genes. (DOC) [file pone.0149843.s004.doc]

**Table S4a.Interaction between rs2066701 of ADH1B and rs1693425 of ADH1C gene**

| **Genotype of rs2066701** | **Genotype of rs1693425** | **Case**  **n=298** | **Control**  **n=162** | **Adjusted OR, 95% CI** | **p-value** |
| --- | --- | --- | --- | --- | --- |
| 11 | 11 | 210 (70.5%) | 138 (85%) | Ref | |
| 22 | 11 | 58 (19.4%) | 18 (11%) | 2.117 (1.2-3.7 ) | **0.012** |
| 22 | 22 | 17 (5.7%) | 3 (2%) | NA | **0.032** |

p<0.05 was taken as significant.

**Table S4b**. Interactions between anti-oxidative genes GSTT1, GSTM1 and rs4880 of MnSOD

| **Genotype of GSTM1** | **Genotype of MnSOD** | **Case**  **n=276(%)** | **Control**  **n=153(%)** | **Adjusted OR, 95% CI** | **p-value** |
| --- | --- | --- | --- | --- | --- |
| GSTM1_WT | 11 | 112 (41) | 89 (58) | Ref | |
| GSTM1_Null | 11 | 92 (33) | 40 (26) | 1.83 (1.15-2.91) | **0.012** |
| GSTM1_Null | 22 | 33 (12) | 6 (4) | NA | **0.001** |
| **Genotype of GSTT1** | **Genotype of MnSOD** | **Case**  **n=279(%)** | **Control**  **n=153(%)** | **Adjusted OR, 95% CI** | **p-value** |
| GSTT1_WT | 11 | 170 (61% ) | 114 (75%) | Ref | |
| GSTT1_Null | 22 | 18 (6%) | 2 (1%) | NA | **0.008** |
| **Genotype of GSTT1** | **Genotype of GSTM1** | **Case**  **n=309(%)** | **Control**  **n=168(%)** | **Adjusted OR, 95% CI** | **p-value** |
| GSTT1_WT | GSTM1_WT | 148 (48) | 108 (64) | Ref | |
| GSTT1_WT  GSTT1_null | GSTM1_null  GSTM1_null | 101 (33)  36(11) | 40 (24)  9(5) | 1.84 (1.18 -2.87)  3 (1.65 3.31) | **0.007**  **0.007** |

p<0.05 was considered as significant

**Table S4C:** Inter gene interaction between ALD metabolism and oxidative stress related genes.

| **Genotype of rs2066701** | **Genotype of rs4880** | **Case**  **n=272(%)** | **Control**  **n=148(%)** | **Adjusted OR,**  **95% CI** | **p-value** |
| --- | --- | --- | --- | --- | --- |
| 11 | 11 | 154(57) | 107(72) | Ref | |
| 11 | 22 | 53(19) | 20 (13) | 1.84 (1.04 -3.26) | **0.041** |
| 22 | 11 | 46 (17) | 17 (12) | 1.88 (1.02-3.46) | **0.044** |
| 22 | 22 | 19 (7) | 4 (3) | NA | **0.043** |
| **Genotype of rs2066701** | **Genotype of GSTM1** | **Case**  **n=300(%)** | **Control**  **n=163(%)** | **Adjusted OR,**  **95% CI** | **p-value** |
| 11 | Wild Type | 126(42) | 99(61) | Ref | |
| 11 | Null | 97(32) | 42 (26) | 1.82 (1.16 -2.84) | **0.011** |
| 22 | Null | 37 (12) | 5 (3) | NA | **<0.001** |
| **Genotype of rs2066701** | **Genotype of GSTT1** | **Case**  **n=304(%)** | **Control**  **n=163** | **Adjusted OR,**  **95% CI** | **p-value** |
| 11 | Wildtype | 181(60%) | 124 (76%) | Ref | |
| 22 | Wildtype | 65 (21%) | 21 (12.5%) | 2.1 (1.22 -3.6 ) | **0.008** |
| 22 | Null | 13 (4%) | 1 (0.5%) | NA | **0.021** |
| **Genotype of rs1693425** | **Genotype of rs4880** | **Case**  **n=275(%)** | **Control**  **n=152(%)** | **Adjusted OR,**  **95% CI** | **p-value** |
| 11 | 11 | 182(66) | 122(81) | Ref | |
| 11 | 22 | 67 (24) | 23 (15) | 1.95 (1.15 -3.31) | **0.013** |
| **Genotype of rs1693425** | **Genotype of GSTM1** | **Case**  **n=276(%)** | **Control**  **n=152(%)** | **Adjusted OR,**  **95% CI** | **p-value** |
| 11 | Wild Type | 143 (52) | 104 (68) | Ref | |
| 11 | Null | 104 (38) | 42 (28) | 1.73 (1.14-2.24) | **0.013** |
| 22 | Null | 16 (6) | 1 (1) | NA | **0.003** |
| **Genotype of rs1693425** | **Genotype of GSTT1** | **Case**  **n=276(%)** | **Control**  **n=152** | **Adjusted OR,**  **95% CI** | **p-value** |
| 11 | Wildtype | 206 (75) | 129 (85) | Ref | |
| 22 | Wildtype | 65 (21) | 21 (12.5) | 2.1 (1.22 -3.6 ) | **0.008** |
| 22 | Null | 7 (3) | 0 (0) | **NA** | 0.049 |

p<0.05 was taken as significant.
