# Supplementary material for: Genetic Association and Gene-Gene Interaction Reveal Genetic Variations in ADH1B, GSTM1 and MnSOD Independently Confer Risk to Alcoholic Liver Diseases in India
Source: PLoS One. 2016 Mar 3;11(3):e0149843. doi: 10.1371/journal.pone.0149843 (PMC4777485; doi:10.1371/journal.pone.0149843)
Supplement: S5 Table — (DOC) [file pone.0149843.s005.doc]

**Table S5**: Univariate analysis of genotypes with severity of liver diseases

| **Loci ID** | **ALC^a^**  **n(%)** | **Steato—hepatitis with fibrosis/com-pensated**  **cirrhosis ^b^**  **n(%)** | **Decom-pensated cirrhosis^c^**  **n(%)** | **Adjusted OR, 95% CI,**  **(a vs.b)** | **p-value**  **(a vs.b)** | **Adjusted OR, 95% CI,**  **(a vs. c)** | **p-value**  **(a vs.c)** |
| --- | --- | --- | --- | --- | --- | --- | --- |
| rs2066701CC | 22 (13) | 36 (31) | 42 (22) | 2.85 (1.57-5.17) | **0.001** | 1.86(1.06-3.27) | **0.04** |
|  |  |  |  |  |  |  |  |
| rs1693425 TT | 6 (4) | 15 (13) | 15 (8) | 2.29 (0.87-6.06) | 0.114 | 0.58 (0.27-1.23) | 0.168 |
| rs4880TT | 24 (16) | 27 (25) | 47 (27) | 1.79 (0.97-3.32) | **0.08** | 2.04 (1.18-3.53) | **0.01** |
|  |  |  |  |  |  |  |  |
| GSTT1 null | 20 (12) | 27 (22) | 34 (18) | 2.1 (1.12-3.96) | **0.024** | 1.60 (0.88-2.91) | 0.139 |
|  |  |  |  |  |  |  |  |
| GSTM1 null | 49 (29) | 56 (47) | 81 (43) | 2.13 (1.30 –3.5) | **0.003** | 1.82 (1.17-2.83) | **0.007** |

p<0.05 was considered as significant.
