# Supplementary material for: Genetic Association and Gene-Gene Interaction Reveal Genetic Variations in ADH1B, GSTM1 and MnSOD Independently Confer Risk to Alcoholic Liver Diseases in India
Source: PLoS One. 2016 Mar 3;11(3):e0149843. doi: 10.1371/journal.pone.0149843 (PMC4777485; doi:10.1371/journal.pone.0149843)
Supplement: S6 Table — (DOC) [file pone.0149843.s006.doc]

**Table S6**: Demographic, biochemical and clinical features of the validation cohort (ALD and NASH patients)

| **Variables** | **ALD Patients**  **(n=100)** | **NASH Patients**  **(n=50)** |
| --- | --- | --- |
| **Epidemiology**  Gender M:F | 100:0 (%) | 100:0 (%) |
| Age, Years  (Mean±SD) | 45.16±7.789 | 40±9.148 |
| BMI (kg/m^2^), Mean±SD | 20.37±5.06 | 28.31±4.137 |
| Ascitis | 40/100 | 0/50 |
| **Laboratory Results**  Total Bilirubin  (mg/dl) (Median, Range) | 1.7 (0.6-18.8) | 0.8±0.3 |
| Albumin(g/dl)  (Mean±SD) | 3.43±0.73 | 4.325±0.3 |
| ALT(u/l)  (Median, Range) | 47.00 (20-179) | 43.50(16-263) |
| AST(u/l)  (Median±SD) | 49.58 (21-220) | 66.00 (18-329) |
| Alkaline Phosphatase (IU/L); Mean±SD | 178 (70-242) | 150 (88-190) |
